# The accelerating pace of amyotrophic lateral sclerosis research

**DOI:** 10.1016/j.ebiom.2025.106079

**Published:** 2025-12-10

**Authors:** eBioMedicine

On Nov 11, 2025, *The New York Times* published an article about a group of young women who had met each other online and travelled to a resort in Cape Cod to meet in person, talk, and have fun. What made this meeting special is that the participants have amyotrophic lateral sclerosis (ALS), a devastating neurodegenerative disease for which there is currently no cure. They were accompanied by their caregivers and brought multiple pieces of equipment needed to support them daily—nevertheless, they cherished their time together. They value their relationship especially because they represent a small group within the ALS community as the disease presents more often in males around the age of 60 years. Irrespective of age and sex though, ALS is an isolating and cruel disease that should be prioritised in biomedical research. Indeed, several studies were published during 2025 that tackled ALS from a number of different angles.

Mechanistic studies add insights in disease aetiology and pathophysiology. Building on extensive literature that has implicated environmental exposures in ALS, Midya et al. in the September 2025 issue of *eBioMedicine* generated an extensive time-series dataset of 17 elements in hair strands from 295 patients with ALS and 96 controls. With sophisticated network analysis, they found that patients with ALS are more likely to have systemic dysregulation and loss of synchronisation in elemental biodynamic patterns, particularly in copper regulation. It's currently unclear if these changes are causative or a result of the disease, but they are certainly intriguing. A review by Chang et al. published in August 2025 in *eBioMedicine* emphasised that neuronal loss, proteinopathy, and volume loss in the hypothalamus might contribute to clinically relevant metabolic, sleep, behavioural, and cognitive changes. Altogether, these findings expand our understanding of ALS from a motor neuron to a multisystem disease under environmental influences.

Only 10–20% of patients with ALS have a causative genetic mutation, but mechanistic experiments focusing on the molecular pathways downstream of the genetic changes can accelerate the identification of biomarkers and potential therapeutic targets that will, in the future, benefit familiar and sporadic cases together. This was the focus of a recent Series in *The Lancet Neurology*: four papers described elegantly the complex pathophysiological changes that ultimately lead to motor neuron injury in patients with mutations in the *SOD1, FUS, C9orf72*, and *TARDPB* genes. The emergence of genetic therapies targeting toxic proteins resulting from the above mutations have generated hope for specific subgroups of patients with ALS. Recognising the urgent need for intervention, in 2023 the US Food and Drug Administration approved tofersen (an antisense oligonucleotide that targets *SOD1* mRNA to reduce the synthesis of SOD1 protein) for patients with *SOD1* mutations based only on observed changes in the biomarker neurofilament light. Multiple recent and ongoing studies are generating clinical data to establish its efficacy.

Biomarker studies in ALS contribute to the diagnosis, prognosis, and identification of likely treatment responders. Integration of artificial intelligence in the data analysis can optimise model performance. In this issue of *eBioMedicine*, McFarlane et al investigate the heterogeneity in the trajectories of the Amyotrophic Lateral Sclerosis Functional Rating Scale (ALSFRS-R, often used as the primary clinical outcome measure in clinical trials), among patients. They trained a fully connected neural network on data from the extensive international ALS dataset (PRECISION ALS) to be able to generate individualised ALSFRS-R forecasts. The model generalised well to an unseen dataset (PROACT) and might facilitate patient recruitment, monitoring, and management in the clinic and in clinical trials.

The severity and nature of ALS lead to complexities in designing and completing randomised clinical trials. For this reason, innovative strategies are needed to accelerate therapeutic discoveries. Feldman et al in *The Lancet Neurology* discussed the use of “digital twins”—simulations of how patients are likely to progress without treatment—and platform trials that assess multiple interventions in parallel. Both reduce significantly the size of (or the need for) control groups, and thus facilitate completion of clinical trials and allow administration of active agents to more patients. Adaptive trials that progress from phase 1 to 2 and 3 involve less bureaucracy and can expedite trials. Furthermore, more than one of these approaches can be combined to maximise benefits, and the experience gained should be transferable to the design of clinical trials for other diseases.

The ultimate goal for everyone involved in the study of ALS is improvement in patient care that leads to higher quality of life and extended survival for patients with ALS. Emerging technologies, from genetic therapies to AI integration, should generate new avenues and expedite discoveries. In *eBioMedicine*, we hope to contribute to these efforts, and we welcome studies that add to our understanding and our ability to intervene in ALS.

